# A common haplotype of *KIAA0319* contributes to the phonological awareness skill in Chinese children

**DOI:** 10.1186/1744-9081-10-23

**Published:** 2014-07-11

**Authors:** Cadmon King-Poo Lim, Amabel May-Bo Wong, Connie Suk-Han Ho, Mary Mui-Yee Waye

**Affiliations:** 1 Croucher Laboratory for Human Genomics, School of Biomedical Sciences, The Chinese University of Hong Kong, Shatin, NT, Hong Kong; 2 Department of Psychology, The University of Hong Kong, Pokfulam, Hong Kong

**Keywords:** KIAA0319, Dyslexia, Chinese population, Phonological awareness

## Abstract

**Background:**

Previous studies have shown that *KIAA0319* is a candidate gene for dyslexia in western populations. In view of the different languages used in Caucasian and Chinese populations, the aim of the present study was to investigate whether there is also an association of *KIAA0319* in Chinese children with dyslexia and/or to the language-related cognitive skills.

**Method and results:**

A total of twenty six single nucleotide polymorphisms (SNPs) were genotyped from three hundred and ninety three individuals from 131 Chinese families. Four of the SNPs have been reported in the literature and twenty two being tag SNPs at *KIAA0319*. Analysis for allelic and haplotypic associations was performed with the UNPHASED program and multiple testing was corrected using permutation. Results indicate that *KIAA0319* is not associated with Chinese children with dyslexia but a haplotype consisting of rs2760157 and rs807507 SNPs were significantly associated with an onset detection test, a measure of phonological awareness (*p*_*nominal*_ *= 6.85 10*^*-5*^ and *p*_*corrected*_ *= 0.0029*).

**Conclusion:**

In conclusion, our findings suggest that *KIAA0319* is associated with a reading-related cognitive skill.

## Introduction

Developmental Dyslexia (DD) is a learning disability that is characterized by difficulties in the acquisition of reading and writing, not related to intelligence, motivation or schooling. It is the most common form of learning disability and affects 5-17% of western populations [1]. Several putative loci (*DYX1 – DYX9*) and candidate genes (*KIAA0319, DYX1C1, DCDC2 and ROBO1*) [2] have been identified in DD including the recent findings of the *MRPL19/C2ORF3* genes of *DYX3* locus and the *GRIN2B* gene [3,4].

More consistent replication of results has been reported for the *KIAA0319* and *DCDC2* genes located at the *DYX2* locus of 6p21–p22. The *DYX2* locus was first refined by high dense SNPs (~21 kp apart) and showed a significantly associated region that contains five genes: *VMP, DCDC2, KIAA0319, TTRAP* and *THEM2*[5]. Francks et al. found a significant region spanning across *TTRAP (TDP2)*, upstream of *THEM2* and the first four exons of *KIAA0319* genes [6]. A risk haplotype consisting of SNPs rs4504469, rs2038137, and rs2143340 was significantly associated with several reading-related measures in two independent samples from Oxford UK and US that were selected for severity of phenotype of DD. Cope et al. also performed a two-stages association study on case–control and trio UK samples that were typically representative of reading disability [7]. After the second stage of stepwise logistic regression analyses, only two SNPs (rs4504469 and rs6935076) located at *KIAA0319* showed highly significant association with DD. Harold et al. further confirmed the association of *KIAA0319* with DD by studying these two independent UK samples (Oxford and Cardiff) together [8]. Five SNPs (rs4504469, rs2179515, rs761100, rs2038137 and rs1555090) were significantly associated with DD in both sets of samples. With the exception of rs4504469, all were clustered around the first exon of *KIAA0319*.

Cell line models have also been studied. Paracchini et al. studied the functional consequences of these sequence variants in lymphoblastoid cell lines [9]. The risk haplotype consisted of rs4504469, rs2038137 and rs2143340, which expressed around 40% lower levels of *KIAA0319* when compared with that of the non-risk haplotype. Dennis et al. further characterized the 5′ upstream of *KIAA0319* and identified a SNP marker rs9461045 that was not only significantly associated with DD and previously reported to be associated with reading-related traits but it was also found by luciferase-based assays that it could influence gene expression, possibly by alteration of the binding site to transcriptional silencer OCT-1 [10]. Therefore, one could possibly conclude that genetic variants influence the functions of *KIAA0319* by alteration of gene expression level.

*In-situ* hybridization of *Kiaa0319* on sections of mouse and human brain indicated that *Kiaa0319* is expressed during mouse and human fetal brain development, and is involved in neuronal migration for formation of the cerebral neocortex [9]. This role was supported by the experiment of *in-utero* RNAi targeting the rat fetal dorsal forebrain. *In-utero* RNAi targeting could inhibit the expression of *Kiaa0319* in the brain cells. The results showed that the neurons failed to associate with the glial fibers and so the initiation of the migration process was affected. These pieces of evidence support the fact that *KIAA0319* may have a role in the development of dyslexia.

Most of the abovementioned findings came from study of Caucasian populations. The role of these genes in Chinese populations had not been reported until the study of Lim et al. [11]. The association of rs3743205 in *DYX1C1* with dyslexia was replicated in Chinese children. This maker was also associated with several cognitive skills including rapid naming, phonological memory and orthographic skills. Therefore, those genetic factors found in other populations could also play important roles in Chinese individuals. This observation led us to propose that KIAA0319 may also contribute in part to the development of dyslexia in Chinese children.

## Materials and methods

### Subjects

In total, 393 individuals from 131 Chinese families were recruited with informed consent. This study was approved by the ethical committee of The Chinese University of Hong Kong. Each family consisted of both parents and one dyslexic child, with a total of 95 males and 36 females, aged between 5 and 16 years (mean age = 8.68 ± 2.06 years). They were diagnosed as DD using the Hong Kong Test of Specific Learning Difficulties in Reading and Writing (HKT-SpLD) [12] and referred by the local education authority, child assessment centres, and a parent association (The Hong Kong Association of Learning Disability). The HKT-SpLD battery consists of 12 subtests. The subtests are divided into three literacy tests, Chinese Word Reading, One-minute Reading and Chinese Word Dictation, and one rapid naming test, where subjects are asked to name digits, colours and pictures as fast as possible. Two subtests are phonological awareness which tests the subjects’ awareness of onsets and rhymes of Chinese syllables, and three phonological memory subtests where subjects are asked to repeat orally the syllables presented to them from a CD player. The final three subtests, Left-Right Reversal, Lexical Decision, and Radical Position, are tests of orthographic skills. The subjects are asked to detect orthographic items in correct orientation, position, or combination.

These 12 subtests were combined to yield five composite scores in the domains of literacy, phonological awareness, phonological memory, rapid naming and orthographic skills. The sample characteristics of these phenotypic measures have been described by Lim et al. [11]. To be classified as children with dyslexia, their literacy composite score and at least one cognitive composite score had to be at least one standard deviation (SD = 3) below the mean (mean = 10) of their respective ages in the HKT-SpLD (cutoff score = 7). Participants in the dyslexic group fulfilled this diagnostic criterion and all of the subjects showed a normal intelligence on Raven’s Standard Progressive Matrices (with IQs of 85 or above). This cohort was also genotyped for variants in the previous study [11].

### SNP markers selection

The tag SNPs selection of *KIAA0319* and *TDP2* was based on previous reports that the significant 3-SNPs haplotype is across these two genes [9] and the functionally significant SNP rs9461045 was forced into the selection [10]. The *KIAA0319-TDP2* region is spanning Chr6: 24659071 to 24545939 (Genome Reference Consortium Human Build 37.1, NT_007592.15). Twenty-four tag SNPs, except rs2038737, were selected using the TAGGER program as implemented in HaploView 4.1 [13] with parameters of minor allele frequency over 5% and pairwise r^2^ threshold of 0.8, based on the population of Han Chinese genotype data generated by the HapMap project (Data Rel#22/phase II Apr 07). The SNP rs2038137 of the 3-SNPs haplotype was not forced into tag SNPs selection because it was not genotyped in the HapMap project.

### DNA extraction and genotyping

Two milliliters of saliva was collected from each individual. Genomic DNA was extracted using the Oragene™ DNA self-collection kit following the manufacturer’s instructions (DNA Genotek, Inc., Ottawa, Canada) and DNA quantity was determined by Quant-iT™ DNA Assay Kit, Broad Range (Invitrogen Corporation, California, USA). Genotyping was performed using Sequenom® MassARRAY® iPLEX Gold assay as described before [11]. Markers were checked for Mendelian inconsistencies and tests of Hardy-Weinberg equilibrium using Pedstats [14].

### Statistical analyses

Family-based and haplotype association analyses were performed using UNPHASED (Version 3.1.2) which employs an allelic likelihood ratio test [15]. Haplotype analysis was performed using the 2- or 3- markers sliding windows method. Initially, a global analysis was performed to test for haplotypic association and then the significant haplotypes were subsequently tested for individual haplotype analysis. Haplotypes with frequencies <1% in the whole sample were excluded. The analysis option of conditioning markers was selected for testing direct association of a single marker in the significant haplotypes. Permutation test (1000 runs) was also used to run multiple testing corrections over all tests performed in single-marker association analyses of categorical DD. Linkage disequilibrium (LD) was calculated and LD plots were generated using Haploview version 4.1 (http://www.broad.mit.edu/mpg/haploview) [13]. For quantitative traits analysis, additive genetic value (AddVal) was estimated using UNPHASED with the value which gives the change in expected trait value due to the haplotype of interest relative to the reference haplotype being selected. AddVal assumes a normally distributed trait and small deviations from the mean.

## Results

### Association of *KIAA0319* with Chinese children with dyslexia

All markers were in Hardy-Weinberg equilibrium (p > 0.1) except rs6901322 which was excluded in this study. Single marker analyses of categorical DD only showed two modestly significantly associated SNPs: rs3756821 (*p = 0.0433*) and rs9366577 (*p = 0.0459*) (Table 1). They could not withstand the multiple testing correction and that the adjusted p value after correction is 0.5794. The haplotype consisting of rs3756821 and rs9366577 was tested but did not show a significant association (*p = 0.06250*) (Table 2). Haplotypic associations were also tested using the sliding-window method (Table 2). Only marginal p values (0.0280 - 0.0375) were obtained and significance was lost after multiple corrections.

**Table 1:** **Single-marker analyses between SNPs of *KIAA0319 *** **and categorical DD**

**rs number**	**SNP**	**Position**	**Location**	**Reference allele (Frequency)**	**OR (95% CI)**	**Nominal p-value**
rs2143340	C/T	24659071	Intron 2 ^#^ (TDP2 gene)	C (0.150)	1.38 (0.86–2.23)	0.1845
rs9461045	C/T	24649061	5′ Upstream	C (0.346)	0.83 (0.57–1.21)	0.3400
rs3756821	C/T	24646821	5′ Upstream	C (0.779)	1.58 (1.01–2.48)	0.0433*
rs2038137	G/T	24645943	Intron 1	G (0.847)	0.89 (0.51–1.54)	0.6743
rs2038139	A/C	24645420	Intron 1	A (0.877)	0.88 (0.49–1.57)	0.6546
rs9366577	C/T	24641328	Intron 1	C (0.046)	0.40 (0.16–1.03)	0.0459*
rs730860	A/T	24632427	Intron 1	A (0.284)	0.95 (0.62–1.46)	0.8273
rs12194307	A/T	24625562	Intron 1	A (0.896)	0.86 (0.47–1.60)	0.6393
rs9467239	C/G	24624857	Intron 1	C (0.398)	0.98 (0.69–1.41)	0.9270
rs6915373	C/T	24612902	Intron 1	C (0.540)	0.90 (0.63–1.30)	0.5806
rs5026394	A/C	24590547	Intron 3	A (0.583)	1.13 (0.78–1.64)	0.5101
rs4504469	A/G	24588884	Exon 4	A (0.129)	1.11 (0.66–1.85)	0.6961
rs9295626	C/T	24587339	Intron 4	C (0.756)	1.09 (0.73–1.61)	0.6861
rs2760135	C/T	24585547	Intron 4	C (0.438)	1.15 (0.72–1.82)	0.5583
rs2817200	A/G	24584366	Intron 4	A (0.774)	0.79 (0.50–1.24)	0.2981
rs6901322	A/T	24583804	Intron 5	Not in Hardy-Weinberg equilibrium (p < 0.1)		
rs699461	A/G	24582862	Intron 5	A (0.733)	1.06 (0.72–1.56)	0.7675
rs807507	C/G	24579867	Intron 8	C (0.215)	1.15 (0.75–1.77)	0.5125
rs2760157	C/T	24578272	Intron 9	C (0.486)	0.98 (0.69–1.41)	0.9276
rs12213545	C/T	24568452	Intron 13	C (0.900)	1.10 (0.61–1.98)	0.7630
rs807540	C/T	24559029	Intron 17	C (0.551)	1.03 (0.72–1.48)	0.9263
rs9467220	C/T	24550964	Intron 20	C (0.074)	0.60 (0.29–1.23)	0.1551
rs10456306	C/T	24550041	Intron 20	C (0.243)	1.05 (0.69–1.59)	0.8312
rs807532	C/T	24549668	Intron 20	C (0.467)	0.95 (0.67–1.36)	0.7868
rs807530	C/G	24545939	UTR-3	C (0.782)	1.13 (0.76–1.68)	0.5443

*Adjusted p-value from permutation test is 0.5794. ^#^This is the only SNP located in *TDP2* gene. All the others are located in *KIAA0319* gene.

**Table 2:** **Haplotype analyses of *KIAA0319 *** **region using 2- or 3-markers sliding windows in association with categorical DD**

**Haplotypes**		**Frequency**	**Global p-values**	**Individual haplotype test**		**Adjusted P value**
				**OR (95% CI)**	**p-values**	**Permutation (1000)**
rs3756821- rs9366577	C-T	0.791	0.0625	1	0.0495	
	T-T	0.168		0.5323 (0.1866 – 1.519)	0.2568	
	T-C	0.040		0.3874 (0.1499 – 1.001)	0.0442	
rs4504469 rs2038137 rs2143340	T-T-G	0.668	0.3579	0.3703 (0.0906 – 1.513)	0.4093	
	T-T-C	0.137		0.2569 (0.05752 – 1.148)	0.1736	
	A-G-G	0.077		0.2598 (0.05393 – 1.251)	0.3173	
	C-G-G	0.065		0.6415 (0.1436 – 2.866)	0.2752	
	A-C-G	0.033		1		
	A-G-C	0.014		0.1851 (0.02041 – 1.68)	0.4142	
**Sliding window**						
**2-markers**						
rs9366577- rs2038139	C-A	0.041	0.0305	1	0.0116	0.3656
	T-A	0.832		0.2667 (0.0885 – 0.8035)	0.3173	
	T-C	0.127		0.2333 (0.0669 – 0.8134)	0.6547	
rs6915373-rs9467239	C-C	0.337	0.0375	1	0.3832	
	C-G	0.202		1.7480 (1.013 – 3.016)	0.0528	
	T-C	0.061		2.4560 (0.9815 – 6.145)	0.0878	
	T-G	0.401		0.9833 (0.6372 – 1.517)	0.1215	
**3-markers**						
rs9366577-rs2038139-rs2038137	C-A-G	0.040	0.02347	5 (1.447 – 17.27)	0.0047	0.3397
	T-A-G	0.788		1	0.2041	
	T-A-T	0.053		1.265 (0.4706 – 3.402)	0.6171	
	T-C-T	0.110		0.7706 (0.374 – 1.588)	0.3692	
**4-markers**						
rs9366577-rs2038139-rs208137- rs3756821	C-A-G-T	0.041	0.0301	5.33 (1.535 – 18.53)	0.0047	0.4306
	T-A-G-C	0.635		1	0.0330	
	T-A-G-T	0.156		1.654 (0.9109 – 3.005)	0.1489	
	T-A-T-C	0.042		1.809 (0.4949 – 6.614)	0.5271	
	T-C-T-C	0.109		0.859 (0.4114 – 1.793)	0.3692	

### Association of *KIAA0319* with reading related traits

**Figure 1:**
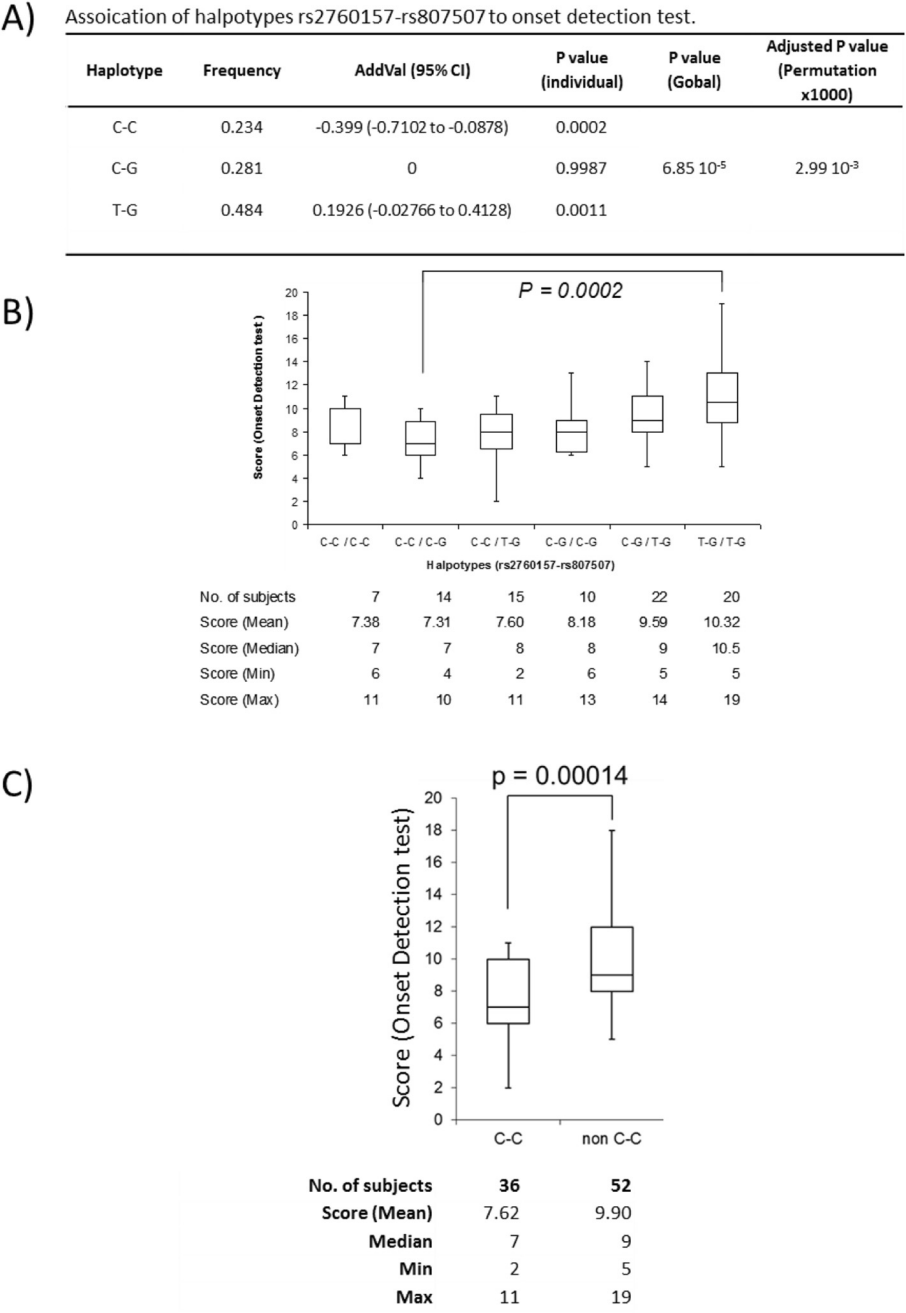
**Haplotype rs2760157-rs807507 associated with onset detection score. (A)** The most significant SNPs, rs2760157 and rs807507, found in single-marker quantitative analyses (onset detection) were used for haplotype analyses. The haplotypes are significantly associated with onset detection. C-C haplotype is the risk haplotype with -0.399 AddVal (95% CI = -0.7102 to -0.0878). **(B)** Plot of possible haplotypes found in each individual shows a decreasing trend of onset detection score for person carrying C-C haplotype. **(C)** The onset detection score of C-C carriers is significantly different from non-C-C carriers.

**Figure 2:**
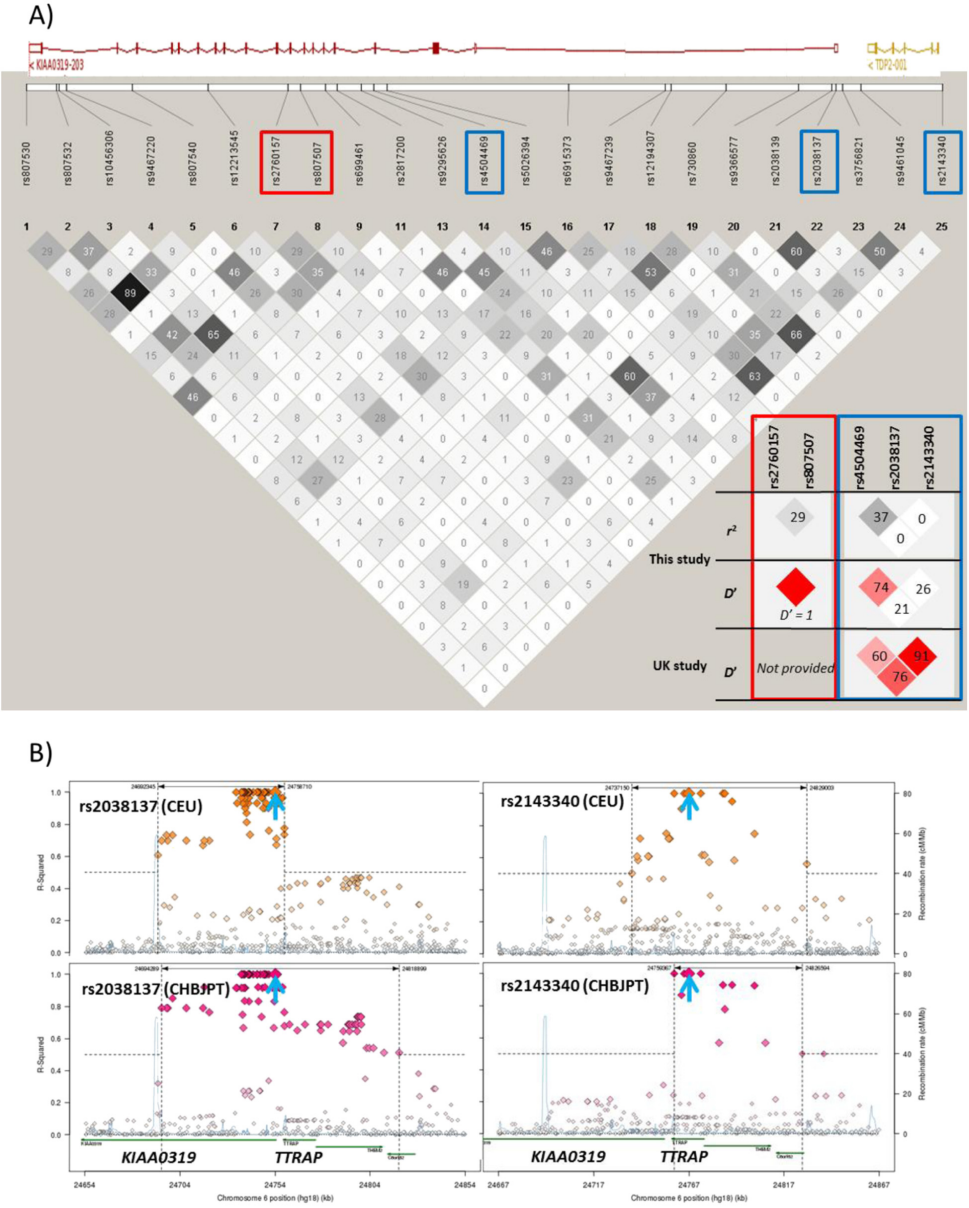
**Linkage disequilibrium plots of SNPs located in *KIAA0319.*(A)** Inter-SNP linkage disequilibrium was generated for studied markers covering *KIAA0319* using Haploview 4.0 [13]. Color scheme represents the *r*^2^ value (stronger linkage disequilibrium is shown using progressively darker shading). Schematic diagram of KIAA0319 and TDP2 genes obtained from Ensembl (http://www.ensembl.org). Right bottom: Linkage disequilibrium plots of the significant haplotype rs2760157-rs807507 (Red box) found in this study (Hong Kong sample) and the haplotype rs4504469-2038137-2143340 (Blue box) studied in Paracchini et al. (UK sample) [16]. Linkage disequilibrium plot represent D’ measure was also generated. The cells are color in red gradually representing strength of LD between the two markers: Bright red (LOD ≥ 2 D’ = 1), pink red (LOD ≥ 2 D’ < 1) and white color for no or weak LD (LOD < 2, D’ < 1). **(B)** Regional LD plots in *KIAA0319* 5′ and *TTRAP* (*TDP2*) genomic region (plots were generated using SNAP web-based software at the Broad Institute [17] and data from 1000 Genomes Project Pilot 1). *r*^2^ value relative to rs2038137 and rs2143340 (indicated by blue arrows) in CEU and CHBJPT populations for SNP in a 100Kb window around it plotted against their genomic location. Dotted vertical lines delimit regions including SNPs with *r*^2^ > 0.5. Size and color intensity of markers are proportional to *r*^*2*^ values.

Nominally significant associations were detected with several traits (Table 3). However, only the SNPs, rs2760157 and rs807507, were still significantly associated with Onset Detection test of Phonological Awareness after permutations. The adjusted p value of rs2760157 was 0.03297 and rs807507 was marginally significant (nominal p value was 0.0013 and the empirical 5% quantile was 0.0012). Using these two SNPs for haplotype analyses, the halpotypes were found to be significantly associated with the onset detection trait (*p*_*nominal*_ *= 6.85 10*^*-5*^ and *p*_*corrected*_ *= 0.0029*) Figure 1(A). The linkage disequilibrium map of the SNPs studied shows that rs2760157 and rs807507 are not in strong linkage disequilibrium (*r*^2^ = 0.296 & D’ =1) (Figure 2A). Three haplotypes rs2760157-rs807507 (C-C, C-G and T-G) were derived from the results. Individual haplotype analyses showed that C-C and T-G were significantly associated with Onset Detection (C-C p = 0.0002 and T-G p = 0.0011) (Figure 1(A)). Correlation between performance of onset detection and haplotypes inherited by children with dyslexia was analyzed (Figure 1(B)). Haplotype C-C was shown to be correlated with poor performance of Onset Detection (lower score) and T-G was correlated with better performance (higher score). The group of children with C-C/C-G was significantly different from children with T-G/T-G (*p = 0.0002*) in onset detection. When all the C-C genotypes (C-C/C-C, C-C/C-G and C-C/T-G) are grouped and compared with other non C-C genotypes (C-G/C-G, C-G/T-G and T-G/T-G), a prominent effect is seen (Figure 1(C)).

**Table 3:** **Quantitative analysis of *KIAA0319 *** **single SNPs in HKT-SpLD tests**

	**Literacy**			**Rapid naming**	**Phonological awareness**		**Phonological memory**			**Orthographic knowledge**		
	**CWR**	**OMR**	**CWD**	**DRN**	**RD**	**OD**	**WRI**	**NWR**	**WRII**	**LRR**	**LDT**	**RP**
rs807530												
rs807532			0.01880								0.03103	
rs10456306						0.04196						
rs9467220										0.01335		0.03065
rs807540			0.00482								0.04384	
rs12213545		0.00646										
rs2760157						0.00070						
rs807507						0.00133						
rs699461												
rs2817200		0.03550								0.03002		
rs2760135												
rs9295626											0.04270	
rs4504469				0.00524								
rs5026394		0.01932									0.00335	
rs6915373											0.01081	
rs9467239												
rs12194307											0.04283	
rs730860	0.00416	0.01054										
rs9366577												
rs2038139				0.01119								
rs2038137			0.04953	0.02454		0.02260						
rs3756821												
rs9461045												
rs2143340												0.01381
Adjusted P value (permutation × 1000)						0.03297						

Only p values < 0.05 are shown. Abbreviations: Chinese Word Reading (CWR), One Minute Reading (OMR), Chinese Word Dictation (CWD), Digit Rapid Naming (DRN), Rhyme Detection (RD), Onset Detection (OD), Word Repetition I (WPI), Non-word Repetition (NWR), Word repetition II (WRII), Left-Right Reversal (LRR), Lexical Decision (LD) and Radical Position (RP).

## Discussion

In this study, the SNPs clustered around the first exon show only nominally significant association with DD. The markers rs3756821 and rs9366577 have nominal p-values, 0.0433 and 0.0459, respectively. Also, the 3-markers (rs9366577-rs2038139-rs2038137) and 4-markers (rs9366577-rs2038139-rs208137-rs3756821) haplotypes show nominal significance and the risk haplotypes (C-A-G and C-A-G-T) have odds ratios around 5. However, both single-marker and haplotype analyses could not withstand the multiple testing corrections. Besides, previous reported SNPs rs4504469 (this study: *p = 0.6961*), rs2038137 (this study: *p = 0.6743*) and rs2143340 (this study: *p = 0.1845*) (Francks et al. [6]) and the haplotypes formed by these SNPs are not associated with DD (this study: *p = 0.3578*). More consistent replication of results has been reported for the region spanning across *TTRAP (TDP2)*, upstream of *THEM2* and the first four exons of *KIAA0319* gene [6-8].

Francks et al. reported that the main risk haplotype (1-1-2 of rs4504469-rs2038137-rs2143340) was associated with several reading traits including orthographic coding test for irregular words (OC-irreg), orthographic coding forced word choice test (OC-choice), single-word reading ability (READ) and spelling ability (SPELL) in the UK sample [6]. The most significant trait was OC-choice (*p = 0.00007*). The main “protective” haplotype, 2-2-1 which has a positive average effect on reading-related measures, was also associated with these reading traits plus the phonological decoding ability (PD) (*p = 0.017*). These results were confirmed by Harold et al. in a study combining two independent UK samples, and the investigators found that the SNPs clustered around exon 1 of *KIAA0319* was associated with reading-related traits [8]. It is not surprising, though, that the significant reading-related traits found were similar to those found by an earlier study as the same UK samples were used in both studies [6]. Again, the OC-choice was the most significant trait. To determine the effect of *KIAA0319* on reading skills, two independent studies investigated the genetic influences of *KIAA0319* in the general population rather than in groups of individuals with reading problems. Paracchini et al. studied the previously reported markers in a sample of 600 7–9-year-old children recruited from the general UK population [16]. The minor allele of rs2143340 marker showed the most significant association with poor performance of reading and spelling measures. The risk haplotype of rs4504469-rs2038137-rs2143340 was shown to be associated with poor performance, and this was consistent with previous reports. However, the haplotype rs4504469-rs6935076 reported by Cope et al. did not show any significant association with phonological related measure (phoneme awareness) [7]. A similar study was conducted in 440 Australian families, looking at the gene *KIAA0319* and its influence on reading skills [18]. However, an opposite trend was observed with the minor allele of rs2143340 and risk haplotype of dyxleisa reported by Francks et al. in that conversely, these haplotypes were associated with better, rather than worse, performance [6].

For quantitative analyses, several markers are nominally associated with traits tested in this study (Table 3). However, only the rs2760157 is still significantly associated with OD after multiple testing correction (adjusted *p = 0.03297*) and the rs807507 is marginally significantly associated with OD for adjusted p value (the empirical 5% quantile is 0.001231). The haplotype consisting of rs2760157 and rs807507 shows highly significant association with OD (nominal *p = 6.85 × 10*^*-5*^ and adjusted *p = 2.99 × 10*^*-3*^). The AddVals show that C-C haplotype has a negative effect on onset detection test (AddVal = -0.399) but T-G shows a positive effect on OD (AddVal = 0.1926) as C-G is a reference haplotype. The results seen in the present study are inconsistent with those in previous reports in that the markers of *KIAA0319* did not associate with dyslexia and the quantitative trait analyses show different associated markers that are not located around the first exon of *KIAA0319*. Also, the onset detection test of phonological awareness, instead of orthographic-related measure OC-choice is the most significantly associated reading skill found in this study that still remained significant after correction for multiple testing.

Disparity may be explained by differences in entry criteria, sample size and study strategies. Differing entry criteria might be a possible explanation for the discrepancy seen in the different studies. For example, Francks et al. recruited the first 192 UK families based on their single-word reading ability below 2SDs and the remaining 72 families based on ability below 1SD [6]. Cope et al. recruited their UK subjects using the inclusion criteria for probands with an IQ larger or equal to 85 and a reading age larger or equal to 2.5 years behind that expected for that chronological age [7]. Brkanac et al. recruited subjects with the proband to have a score at least 1SD below that of the population mean on at least one of the 10 reading tests [19]. Our inclusion criteria were based on the Hong Kong Test of Specific Learning Difficulties in Reading and Writing (HKT-SpLD) [12]. The children were classified as suffering from dyslexia if their literacy composite score and at least one cognitive composite score were at least one SD below the means of their respective ages. These criteria are different from other studies; for example, only literacy (single-word reading ability) was used to assess children in Francks et al. [6].

Sample size and study strategies may be the other reasons for the variation in association results. Francks et al. studied 264 nuclear families [6], Cope et al. combined several approaches: DNA pooling, case–control and trio family [7] and Brkanac et al. studied 191 families but only two previously reported SNPs were used [19]. In this study, 131 trios were used, but the sample size is smaller, compared to that of Francks et al. [6], the small sample size may pose a problem of insufficient sample power.

Besides, although association of the risk haplotype was replicated in several studies [6,8,16], these SNPs may not be the true causative variations for dyslexia or reading-related phenotypes. The haplotype spans 70 k bps from intron 1 of *TAP2* (rs2143340) to exon 4 of *KIAA0319* (rs4504469). These markers may be in linkage disequilibrium with the true causative variation within or near this region. The study of gene expression indicated that only expression of *KIAA0319* but not *TAP2* was lower in cell lines which carried the risk haplotype [9]. Therefore, rs2143340 may not have a functional role in the regulation of *KIAA0319*. Recently, Dennis et al. further characterized the 5′ upstream of *KIAA0319* and identified a SNP marker rs9461045 that was not only significantly associated with DD and previously reported reading-related traits but could also influence the gene expression in luciferase-based assays possibly by alteration of the binding site to transcriptional silencer OCT-1 [10]. These findings lend support to the theory that the true putative variant could be just in LD with the haplotype markers. However, our results did not show any significance of rs9461045 in our sample. This difference could be due to differences in the ethnic origins of our subjects from European.

The linkage disequilibrium of these markers (rs4504469-rs2038137-rs2143340) between the UK sample and this study shows that LD structures are different (Figure 2A). These markers are not in such high LD as those seen in the UK samples [16]. To examine this LD structures in an independent sample, the *r*^2^ values using data from the 1000 Genomes Project Pilot 1 (CEU and CHB + JPT populations) relatives to rs2038137 and rs2143340 for all the SNPs in a 100 kb region around against their genomic location were plotted. In the CEU population, the delimit regions of rs2038137 and rs2143340 with *r*^2^ > 0.5 with other SNPs are different from the Chinese population. Our finding is similar to this sample that rs2143340 is less highly linked with other SNPs in the region than the CEU sample. Paracchini et al. also pointed out the difference of LD structures between four populations: European (CEU), Chinese (CHB), Japanese (JPT) and Yoruban (YRI) [16]. The high LD of rs2143340 to other markers in the 5′ promoter region of *KIAA0319* seems to be unique in European populations.

On the other hand, population admixture in the study of Luciano et al. [18] and Couto et al. [20] could partially explain the inconsistent results. The samples consisted of about 82% Anglo-Celtic ancestry [18] and 68% European or British ancestry [20] respectively. Paracchini et al. also observed that the significance was decreased when the sample included non-white European ancestry [16]. Therefore, the significant findings of rs4504469-rs2038137-rs2143340 could not be replicated in this study may reflect the population difference in LD structure of the 5′ region of *KIAA0319*.

The finding of an association between haplotype (rs2760157-rs807507) and onset detection of phonological awareness is the most significant result in this study. However, no single marker or haplotype (including the rs2760157-rs807507, *p = 0.759*, data not shown) is significantly associated with DD. This may be due to the fact that orthographic knowledge and rapid digit naming, as opposed to phonological awareness are the main deficits in Hong Kong Chinese DD. The gene only shows nominal association with orthographic knowledge (smallest nominal *p = 0.00335* in rs5026394 associated with Lexical Decision (LDT)). Therefore, there is insufficient statistical power to detect the effect in this sample where only minority DD children have problems in phonological awareness. It is interesting to note that phonological awareness is the core deficit in Caucasian populations reading alphabetic scripts, but orthographic skill (OC-choice) was strongly associated with *KIAA0319* in European samples [6,8,10,16]. In addition, the effect sizes associated with these markers are relatively small in these studies. Therefore, it implies that the major genetic factors affecting phonological awareness are still to be defined. However, it should be noted that English orthographic skills cannot be fully dissociated from the phonological component. Confounding phonological legality in judging orthographic legality may be the cause of these associations. Therefore, this observation should be further confirmed by independent testing.

Through studying the reading skills of Chinese–English bilingual children, cross-linguistic transfer was observed in people who have learning difficulties in the first language who would show similar deficits in learning the second language. Chinese onset awareness test is correlated with English word reading tests [21,22]. Both studies also indicate that onset detection is a predictor for English real word reading; however, English orthographic choice does not predict Chinese word reading. Therefore, we could hypothesize that individuals who carry risk haplotype (rs2760157-rs807507) of *KIAA0319* might show more problems learning English than Chinese. To prove this hypothesis, we could test the association of this haplotype to their English reading skills for our subjects.

Pleiotropic effects may be another reason why association with different reading-related traits was seen in Chinese Hong Kong and UK populations. Several studies have reported the dyslexia putative loci were also associated with other language disabilities, such as speech-sound disorder, language impairment and attention-deficit/hyperactivity disorder [23-25]. In particular, pleiotropic effect of *KIAA0319* was found between special language impairment (SLI) and DD. Rice et al. studied some previously reported DD or speech-sound disorder putative loci in SLI subjects [26]. Linkage analyses supported that chromosome 6p22 was linked to several language measures. SNPs of *KIAA0319* including some previously reported SNPs in DD is indeed also associated with the reading measures. Another independent study supported the theory that KIAA0319 has pleiotropic effects. *KIAA0319* was found to be associated with reading measures in both DD and SLI cohorts but was only associated with language measures in the SLI cohort [27]. Therefore, *KIAA0319* could affect phonological awareness and/or orthographic knowledge depending on mutation events arising in different populations.

We may, therefore, reasonably conclude that *KIAA0319* may not be directly associated with dyslexia in Chinese children with the Chinese language being used as the testing criteria, phonological awareness and may influence other language-based disabilities.

## Competing interests

The authors declare that they have no competing interests.

## Authors’ contributions

MW designed the study and supervised the overall experimental part of the project, communicated with the Association of Specific learning disability for help with recruitment of the subjects. CH supervised gathering the reading and writing performance and development of classification schemes for the dyslexic children, and communicated with Government departments and other agencies to obtain details of the phenotypes. CL designed and performed all the genotype analyses and association analyses of risk alleles. AW performed the DNA extraction and genotyping. All authors discussed the results and implications and commented on the manuscript at all stages.
